# Traces of Elements in the Electrochemical Reductive Amination of Acetone: Uncovering Bi as Substitute for Pb

**DOI:** 10.1002/anie.202521065

**Published:** 2026-02-10

**Authors:** Justus Kümper, Yani Guan, Simran Kumari, Sonja D. Mürtz, Philippe Sautet, Regina Palkovits

**Affiliations:** ^1^ Chair of Heterogeneous Catalysis and Technical Chemistry RWTH Aachen University Aachen Germany; ^2^ Department of Chemical and Biomolecular Engineering University of California Los Angeles California USA; ^3^ Department of Chemistry and Biochemistry University of California Los Angeles California USA; ^4^ Institute for a sustainable Hydrogen Economy (IHE‐2) Forschungszentrum Jülich Jülich Germany; ^5^ Max Planck Fellow Max‐Planck‐Institute For Chemical Energy Conversion Mülheim an der Ruhr Germany

**Keywords:** bismuth, electrochemistry, impurities, lead, reductive amination

## Abstract

Amines are broadly utilized as solvents, pharmaceuticals, herbicides, or materials. A benign synthesis route to produce amines from carbonylic substrates is the electrochemical reductive amination, whereby electrons combined with a green proton source like water serve as formal reducing agent. Surprisingly, investigating various p‐block elements as mediator for the electrochemical conversion of acetone in presence of methylamine revealed that only elements of the sixth period show an activity. Building up on these findings and the general low toxicity of Bi compared to Tl or Pb, the electrochemical hydrogenation of *N*‐methylpropan‐2‐imine was optimized by studying the effect of Bi concentration, reaction temperature, and cathode material. Thus, this work highlights Bi as innovative mediator for the electrochemical reductive amination, whereby in presence of a few ppm‐amounts of Bi at ambient temperatures high amine yields are achievable.

## Graphical abstract

This study investigates the impact of various p‐block element impurities in the electrochemical reductive amination and reveals with Bi a less toxic alternative for Pb. The utilization of Bi as additive was further optimized by studying the impact of additive concentration, reaction temperature, and cathode material on amine yield, offering new possibilities to synthesize pharmaceuticals sustainably.

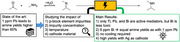

## 1 Introduction

Amines are a versatile class of molecules and play a crucial role in everyday life [1, 2, 3, 4, 5]. Over the years, various synthesis strategies have been developed for the production of amines, with reductive amination being the most commonly used method [6, 7, 8, 9, 10]. The production of secondary amines using reductive amination proceeds via two steps: initially, a condensation reaction takes place between a carbonylic compound and a primary amine forming the corresponding imine. The imine is then reduced to the amine, whereby the nitrogen is fixed in its new molecular structure [3, 11, 12, 13]. Typical hydrogenation processes are either based on molecular reducing agents such as sodium borohydride (NaBH_4_) or molecular hydrogen combined with a catalyst [14, 15]. While the use of NaBH_4_ is characterized by a low atom efficiency and the formation of waste in stoichiometric amounts [14, 16], utilizing hydrogen often depends on harsh reaction conditions like high pressures and/or elevated reaction temperatures [3, 11, 17, 18, 19]. Additionally, the application of molecular hydrogen requires an infrastructure for the production, storage, and transportation of the gas, lowering its attractiveness [14, 20]. In contrast, a sustainable and decentralized hydrogenation of unsaturated bonds is achievable via electrolysis combining electricity from green sources like solar or wind power plants with an environmentally friendly proton source like water [14, 18, 21, 22]. In general, the electrochemical hydrogenation (e‐hydrogenation) of imines in alkaline aqueous media (pH>10) competes with the reduction of the carbonylic compound and hydrogen evolution. As the e‐hydrogenation of imines is favored over carbonyl reduction, only the hydrogen evolution reaction (HER) needs to be suppressed to achieve high Faraday efficiencies [16, 23, 24, 25]. We recently demonstrated that using cathode materials with a high HER overpotential, such as Ag or boron‐doped diamond (BDD), alone does not result in high amine yields. However, with these high‐overpotential cathode materials amine yields exceeding 60% were possible when at least 1 ppm Pb was present in the substrate solution [26]. Lucas et al [27]. studied the e‐hydrogenation of levulinic acid to 4‐hydroxyvaleric acid via in situ attenuated total reflectance Fourier transform infrared (ATR‐FTIR) spectroscopy at a Pb cathode and measured a signal that was assigned to PbH_2_. As a result, the authors assumed a possible participation of PbH_2_ as hydride transfer agent in the e‐hydrogenation of levulinic acid [27]. Interestingly, Pb is not the only element that can be converted into hydride species electrochemically [27, 28, 29, 30, 31, 32, 33, 34, 35]. Under cathodic conditions, As, Se, Sn, Sb, Te, Tl, and Bi can also form volatile hydrides [31, 32, 33, 34, 35]. So far, electrochemical hydride synthesis has mainly been used in trace element analysis to test samples more cost‐effectively and sensitively [30, 31, 32, 34], but it has not yet been used to reduce unsaturated organic molecules. Based on the current state of the art, we hypothesized that other element additives in the ppm‐range could perform equally well in electrochemical reductive amination. Indeed, this study reveals the potential of Bi as an alternative for toxic Pb.

## 2 Results and Discussion

### 2.1 Identifying Active Mediators

**SCHEME 1:**
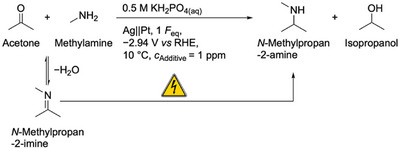
Reaction conditions for the electrochemical reductive amination of acetone in presence of various element additives. The reaction conditions were taken from our previous work [26]. Acetone and methylamine were dissolved in a 0.5 M KH_2_PO_4_ solution (pH: 8.3), which was utilized as solvent and electrolyte. The final substrate solution contained 2.4 M acetone and 2.9 M methylamine with a final pH of 12.9 at 10 °C. The cathode and anode compartments were separated by a N‐424 membrane. The anode compartment contained a 25% H_3_PO_4_ solution. The Ag electrode was the cathode, while Pt was the anode.

**FIGURE 1:**
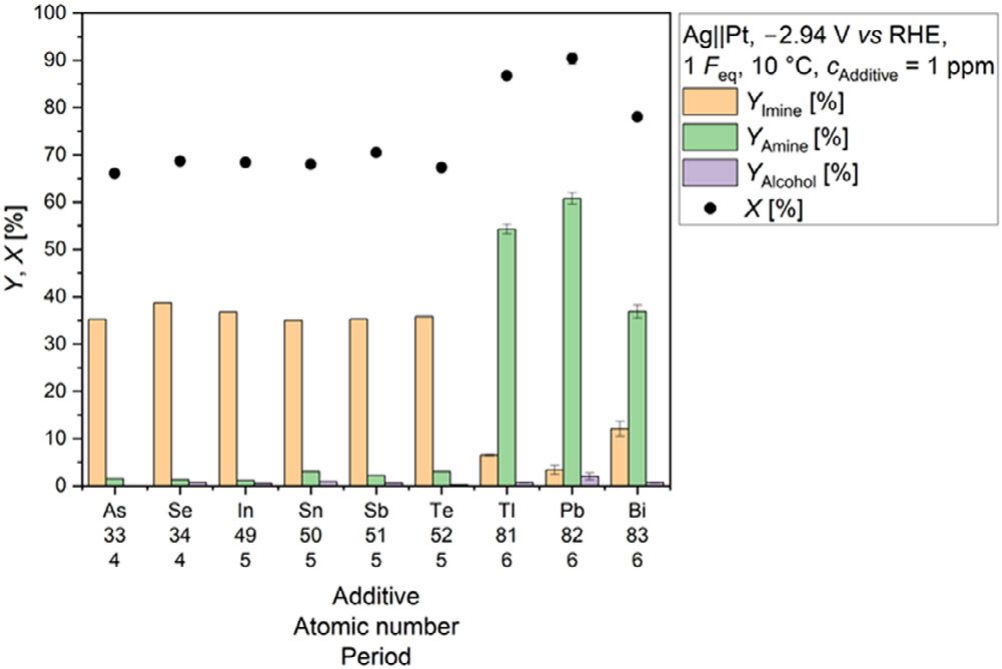
Variation of the element additive and its impact on the electrochemical reductive amination of acetone with methylamine. The final concentration of the additive in the reaction solution before electrolysis was 1 ppm. The yields of *N*‐methylpropan‐2‐imine (*Y*_Imine_), *N*‐methylpropan‐2‐amine (*Y*_Amine_), and isopropanol (*Y*_Alcohol_) are displayed. Furthermore, the conversion of acetone (*X*) is visualized. Carbon balance (CB): 66%–75%. Conditions: Ag||Pt; *E* = −2.94 V *vs* RHE; *F*_eq_ = 1; solvent: 0.5 M KH_2_PO_4_ (pH 8.3); substrates: acetone: 2.4 M, methylamine: 2.9 M; *T* = 10 °C; pH at 10 °C: 12.9; anolyte: 25% H_3_PO_4_; and a N‐424 membrane. The results based on 1 ppm Pb were already published in our previous work [26, 36].

To identify alternative mediators for the electrochemical reductive amination of acetone with methylamine, we tested As, Se, Sn, Sb, Te, Tl, and Bi, since these elements are known to form volatile hydrides under cathodic conditions [31, 32, 33, 34, 35]. Although the electrochemical hydride formation of In has not yet been discussed in the literature, we included it into our study due to its known ability to form volatile hydride species [30]. After electrolysis applying the conditions shown in Scheme 1 and in presence of 1 ppm of the elements As, Se, In, Sn, Sb, and Te, amine yields between 1% and 3% were obtained (Figure 1). This corresponds approximately to that of the Pb‐ and additive‐free system discussed in our previous work [26] (*Y*_Amine_ = 0 ppm Pb [26, 36]: 1.8% (±0.7%)). Accordingly, it cannot be said that these additives have a positive influence on the reductive amination. An explanation for the low yield using Se or Te as additive can be extracted from their Pourbaix diagrams: under alkaline and cathodic conditions, Se and Te form a different type of hydride than the other elements, meaning they exist in their deprotonated form as Se^2−^ or Te^2−^ [37]. This is also supported by the electronegativity of the elements. For Se and Te, the electronegativity difference to H (ΔEN) is ≥ −0.1, while for the other elements ΔEN is ≤ −0.1 [38]. So far, no explanation can be given why As, In, Sn, and Sb show almost no activity as mediator in the electrochemical imine reduction. Since the alcohol yield was less than 0.9% and the amine yield was not higher than 3%, it can be concluded that mainly HER took place in the presence of As, Se, In, Sn, Sb, and Te.

A different picture emerged when the substrate mixture contained 1 ppm Tl or Bi. In the presence of 1 ppm Tl, an amine yield of 54.3% (±1.0%) was obtained, while in the presence of 1 ppm Bi 36.9% (±1.4%) was generated. In both cases, slightly less amine was obtained after application of one Faraday equivalent (*F*_eq_) than using 1 ppm Pb [26, 36] (Tl: −6%; Bi: −24%), but a clearly positive effect was observed compared to the Pb‐ and additive‐free system [26]. In particular, the electrochemical reductive amination was only possible with Tl, Pb, and Bi.

**FIGURE 2:**
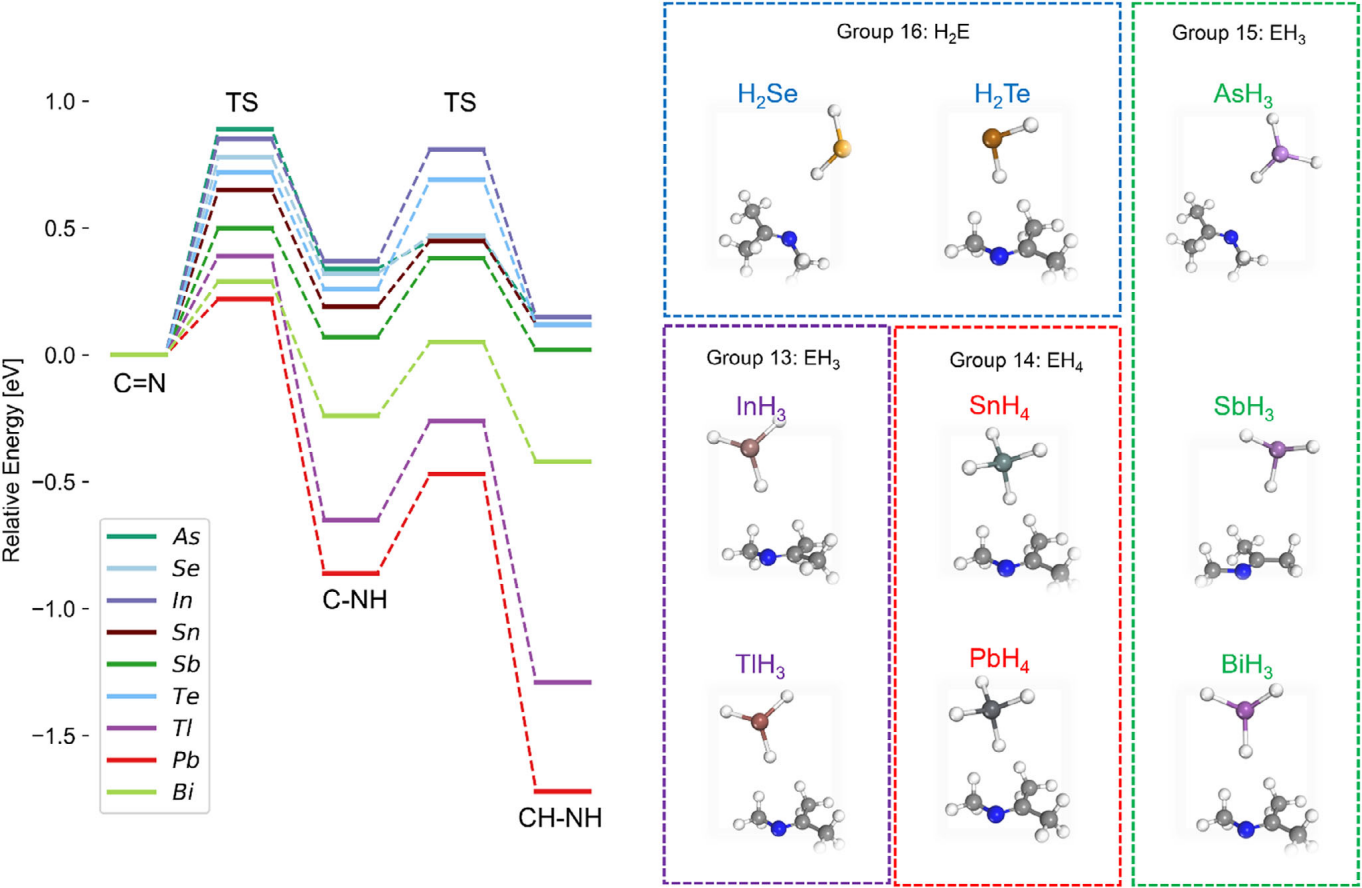
DFT‐calculated energy profiles for the reductive amination of *N*‐methylpropan‐2‐imine to *N*‐methylpropan‐2‐amine mediated by different p‐block element hydrides (AsH_3_, H_2_Se, InH_3_, SnH_4_, SbH_3_, H_2_Te, TlH_3_, PbH_4_, BiH_3_). Two consecutive hydrogen transfer steps are shown, together with the energies of the corresponding transition states (TS). The molecular structures on the right illustrate the initial reactant complexes formed between each hydride and the imine substrate.

To ensure chemically meaningful modeling, we represented each p‐block element by its most characteristic hydride species, reflecting the common oxidation state and coordination preference of the element. Group 14 congeners (Sn, Pb) were modeled as tetrahydrides (EH_4_), consistent with their stable +4 oxidation state. Group 15 elements (As, Sb, Bi) were modeled as trihydrides (EH_3_), corresponding to their predominant +3 state stabilized by the inert‐pair effect. Group 16 elements (Se, Te) were taken as dihydrides (H_2_E), which represent the typical −2 state. For group 13 elements, In and Tl were modeled as EH_3_ (+3) (Figure 2). The calculations clearly reveal that only the sixth period elements Tl, Pb, and Bi provide a downhill energy landscape that facilitates hydrogen transfer, while lighter congeners (As, Se, In, Sn, Sb, Te) remain trapped in energetically unfavorable monohydrogenated intermediates. Effective activation energies and associated kinetics are very favorable for Tl, Pb, and Bi. Consequently, the sixth period elements provide an optimal bond‐strength for the hydride species to efficiently reduce the imine.

### 2.2 Optimizing the Utilization of Bi as Mediator

Even though amine yields of more than 54% have been obtained in the presence of 1 ppm Tl or Pb [26, 36], a potential industrial application using these two additives could prove difficult, especially for the production of pharmaceuticals, as both elements belong to the group of the most toxic metals, with Tl being even more toxic than Pb [39]. In contrast, Bi is classified as largely non‐toxic and is used in cosmetics and pharmaceuticals [40, 41, 42, 43, 44]. Therefore, a residual of ppm‐amounts of Bi in the product mixture after electrolysis could be acceptable. Nevertheless, the comparatively low amine yield of 36.9% (±1.4%) obtained after electrolysis in presence of 1 ppm Bi shows potential for improvement, as around 22% of the acetone and 12% of the imine were still available after electrolysis. Thus, approximately 62% of the current used was consumed for the HER, emphasizing the need to suppress HER.

**FIGURE 3:**
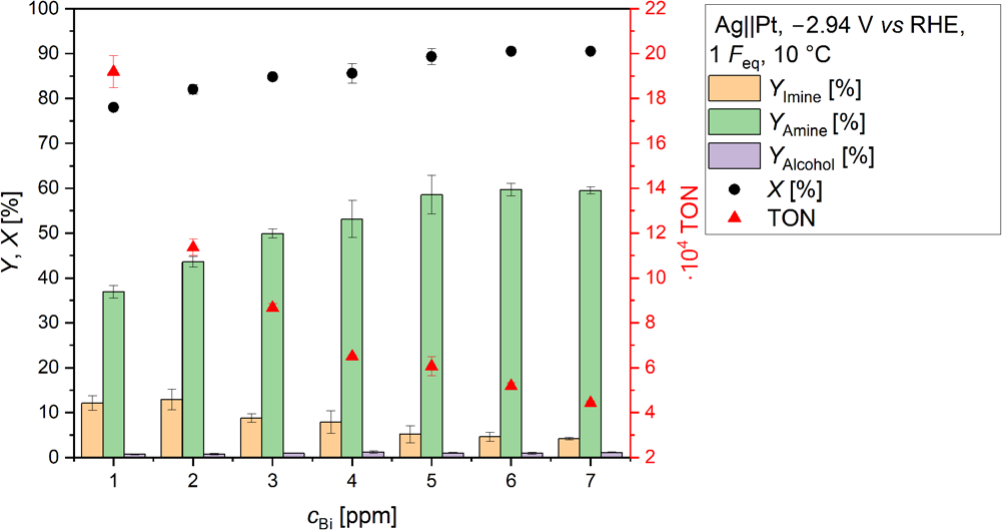
Impact of the Bi concentration on electrochemical reductive amination of acetone with methylamine. The *x*‐axis displays the final Bi concentration in the substrate solution before electrolysis. The yields of *N*‐methylpropan‐2‐imine (*Y*_Imine_), *N*‐methylpropan‐2‐amine (*Y*_Amine_), and isopropanol (*Y*_Alcohol_) are displayed. Furthermore, the conversion of acetone (*X*) and the turnover number (TON) are visualized. Carbon balance (CB): 70%–75%. Conditions: Ag||Pt; *E* = −2.94 V *vs* RHE; *F*_eq_ = 1; solvent: 0.5 M KH_2_PO_4_ (pH 8.3); substrates: acetone: 2.4 M, methylamine: 2.9 M; *T* = 10 °C; pH at 10 °C: 12.9; anolyte: 25% H_3_PO_4_; and a N‐424 membrane.

Consequently, the Bi concentration was varied to identify the lowest amount of Bi required to achieve equal amine yields as with Pb (Figure 3). Increasing the Bi concentration from 1 to 6 ppm in 1 ppm increments led to a continuous raise in amine yield from 36.9% (±1.4%) to 59.7% (±1.4%). In parallel, the conversion increased while the amount of imine decreased, which is in line with expectations. The use of 7 ppm Bi provided no further added value compared to 6 ppm Bi. Interestingly, the alcohol yield was constantly low (*Y*_Alcohol_ ≤ 1.1%) and showed no trend in contrast to the concentration series with Pb as mediator [26].

The generally low alcohol yield indicates that only HER limits or influences the Faraday efficiency of imine reduction in the presence of Bi. Finally, the increasing amine yield with increasing Bi concentration confirmed that the HER can be suppressed by higher Bi concentrations. A disadvantage could be the decreasing average reduction efficiency of the mediator with increasing concentration, which is indicated by the declining turnover number (TON) calculated based on the amount of Bi added to the substrate solution before electrolysis. The decline in TON is due to a smaller raise in the amine yield compared to the change in Bi concentration. It is possible that the active Bi species is formed electrochemically faster than it initiates the reduction. This would lead to a depletion of the inactive Bi species at the electrode, making side reactions such as HER more likely. A raise in Bi concentration would counteract its depletion at the electrode, leading to a stronger suppression of the HER, assuming electrochemical formation of active Bi species is favored over HER. If the average number of inactive Bi species in the system increases with the aim of suppressing the HER, this ultimately leads to a decrease in TON with a simultaneous increase in the Faraday efficiency of imine reduction, which was ultimately also observed. Raising the concentration to 6 ppm not only successfully reduced the HER, but also realized identical amine yields and Faraday efficiencies to those obtained with 1 ppm Pb (*Y*_Amine_ = 6 ppm Bi: 59.7% (± 1.4%); 1 ppm Pb [26, 36]: 60.8% (±1.2%)). Although a six times higher Bi concentration was required for this, the quantities are still trace amounts. On average, one Bi atom still initiated more than 51 000 imine reductions under those conditions, which means that the non‐toxic Bi can be regarded and used as an adequate substitute for toxic Pb.

To gain a mechanistic insight into the role of BiH_3_ species in imine reduction, we computed free energy profiles for two possible hydrogenation pathways by hydrogen transfer (Figure S2). The pathway starting with hydrogenation at the C atom (C═N→CH–N→CH–NH, orange pathway in Figure S2) involves a higher barrier of 0.56 eV and is thus kinetically disfavored. In contrast, the stepwise hydrogenation of the imine bond starting with hydrogen transfer to the N atom (C═N→C–NH→CH–NH, green pathway in Figure S2, also shown in Figure 2) proceeds with a lower barrier (0.29 eV) and results in an overall downhill energy profile (Δ*G* = −0.24 eV), making it the preferred pathway. The formed BiH can be electrocatalytically reduced into BiH_3_ at the Ag electrode after adsorption and BiH_3_ can detach from the surface into solution closing the cycle (Figure S3). This analysis indicates that BiH_3_ does not act as a classical hydride donor, but rather as a hydrogen shuttle between electrode and substrate. Computational results rationalize the experimental observation that Bi can effectively mediate electrochemical reductive amination, albeit with lower activity compared to Tl or Pb.

**FIGURE 4:**
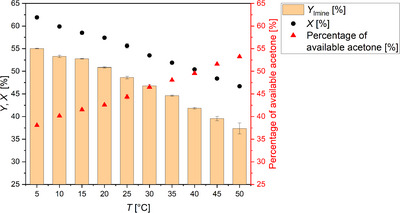
Impact of the temperature on the formation of *N*‐methylpropan‐2‐imine (*Y*_Imine_) and the conversion of acetone (*X*). In addition, the percentage of available acetone is visualized. No electrolysis was performed. Carbon balance (CB): 92%–96%. Conditions: solvent: 0.5 M KH_2_PO_4_ (pH 8.3); substrates: acetone: 2.4 M; methylamine: 2.9 M.

**FIGURE 5:**
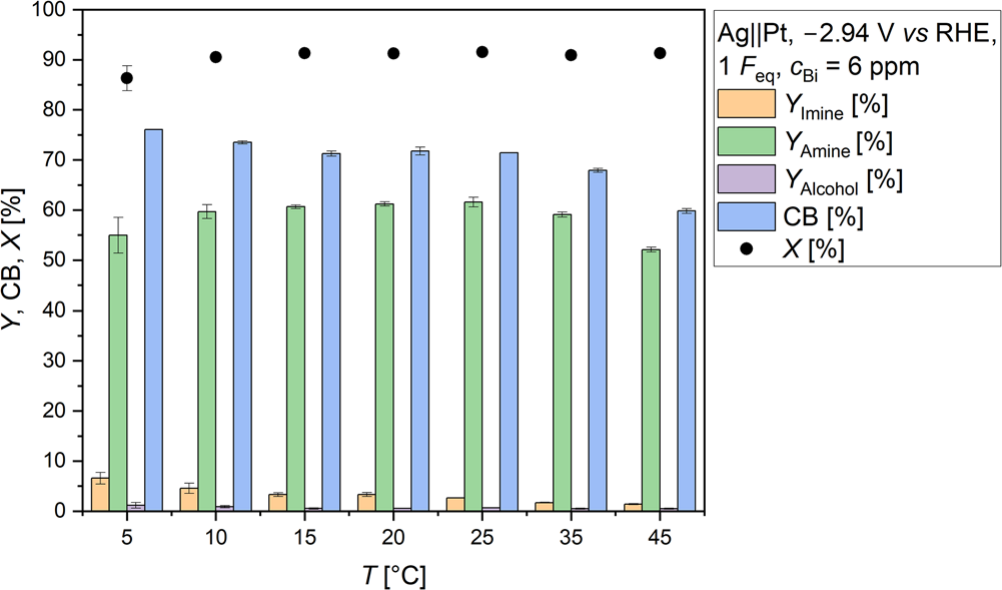
Effect of the temperature on the electrochemical reductive amination in presence of 6 ppm Bi. Yields of *N*‐methylpropan‐2‐imine (*Y*_Imine_), *N*‐methylpropan‐2‐amine (*Y*_Amine_), and isopropanol (*Y*_Alcohol_) are displayed. Furthermore, the conversion of acetone (*X*) and the carbon balance (CB) are visualized. Conditions: Ag||Pt; *E* = −2.94 V *vs* RHE; *F*_eq_ = 1; solvent: 0.5 M KH_2_PO_4_ (pH 8.3); substrates: acetone: 2.4 M, methylamine: 2.9 M; anolyte: 25% H_3_PO_4_; a N‐424 membrane; and 6 ppm Bi in the final substrate solution. pH values of the reaction solution at the different temperatures: 5 °C: 13.1, 10 °C: 12.9, 15 °C: 12.7, 20 °C: 12.4, 25 °C: 12.2, 35 °C: 12.0, 45 °C: 11.4.

Optimization of the reductive amination with Bi as mediator was continued by investigating the influence of electrolysis temperature on product formation. Smirnov and Tomilov [25] observed that formation of the imine is decisive for the success of electrochemical reductive amination, meaning that a favored imine formation leads to higher amine yields [25]. Various influencing variables, such as molecular structures of the substrates and pH value of the electrolysis solution, were considered and correlated with imine and amine formation [25]. However, effects of the electrolysis temperature on imine and amine formation have not yet been investigated. Nonetheless, the temperature has a decisive influence on the equilibrium between *N*‐methylpropan‐2‐imine and the substrates acetone and methylamine (Figure 4). Increasing the temperature from 5 °C to 50 °C resulted in a decrease in imine yield by almost 18% from 55.0% (±0.1%) to 37.4% (±1.2%). Based on these data and the current state of research, it could be expected that higher amine yields would be generated at lower electrolysis temperatures. However, this is only partially true (Figure 5).

Raising the electrolysis temperature from 5 °C to 20 °C or 25 °C resulted in a slight increase in amine yield by 7% to approximately 62%. In contrast, temperatures higher than 25 °C caused a decline in yield (*Y*_Amine_ = 35 °C: 59.1% (±0.5%); 45 °C: 52.1% (±0.5%)). The reduction in amine yield when using higher electrolysis temperatures than 25 °C was probably not a result of the equilibrium shift, but it is more likely that evaporation of organic molecules was the cause. By increasing the electrolysis temperature from 25 °C to 45 °C, the carbon balance decreased from 71.4% (±0.0%) to 59.8% (±0.5%), a decline of almost 12%. For comparison: the amine yield decreased by approximately 10%. The loss in carbon balance can be attributed to the use of acetone and methylamine as substrates, which are very volatile molecules (Table S1). In addition, the electrochemically formed amine has a boiling point of only 49 °C–51 °C [45], which is just 4 °C–6 °C below the electrolysis temperature of 45 °C. Since the amine yield has diminished to a similar extent as the carbon balance, the decreasing yield at higher temperatures can be attributed to evaporation. The use of a lower electrolysis temperature than 20 °C also did not have a positive effect on the amine yield, although this could have been expected with reference to Figure 4. Two options can explain the observed decline: either the hydride formation deteriorates with decreasing temperature or the reactivity of the hydride declines with decreasing temperature. Amberger [46] pointed out that bismuthane (BiH_3_) is a thermally very unstable molecule and can therefore only be produced at low temperatures [46]. At room temperature, it would already decompose [46, 47, 48]. According to these statements, a lower electrolysis temperature should not counteract hydride formation. However, since a decrease in the amine yield was observed, it is more likely that the reactivity of the active Bi species is reduced with lowering electrolysis temperature. Amine yields of more than 60% were obtained for 15 °C, 20 °C, and 25 °C with very good reproducibility. Accordingly, there is no need to cool the electrolysis cell. The yield of the by‐product isopropanol was low regardless of the temperature, as a higher yield than 1.2% (±0.6%) was not obtained. This shows that Bi is a selective mediator for imine reduction, as the percentage of free acetone generally increased with electrolysis temperature (Figure 4). At an electrolysis temperature of 45 °C, for example, the initial imine yield was 39.6% (±0.5%), while the proportion of unreacted acetone was 51.6% (±0.1%). Thus, more acetone than imine was present at the beginning, but less than 1% alcohol was obtained after electrolysis. This illustrates that the equilibrium between the intermediate and the substrates is dynamic and the imine formation seems not to be the kinetically limiting step as the final amine yield was equal to or higher than the initial imine yield (cf. Figure 4 with Figure 5). Accordingly, the depletion of the imine as a result of the irreversible hydrogenation provoked the formation of further imine molecules, which is consistent with Le Chatelier's principle [48].

**FIGURE 6:**
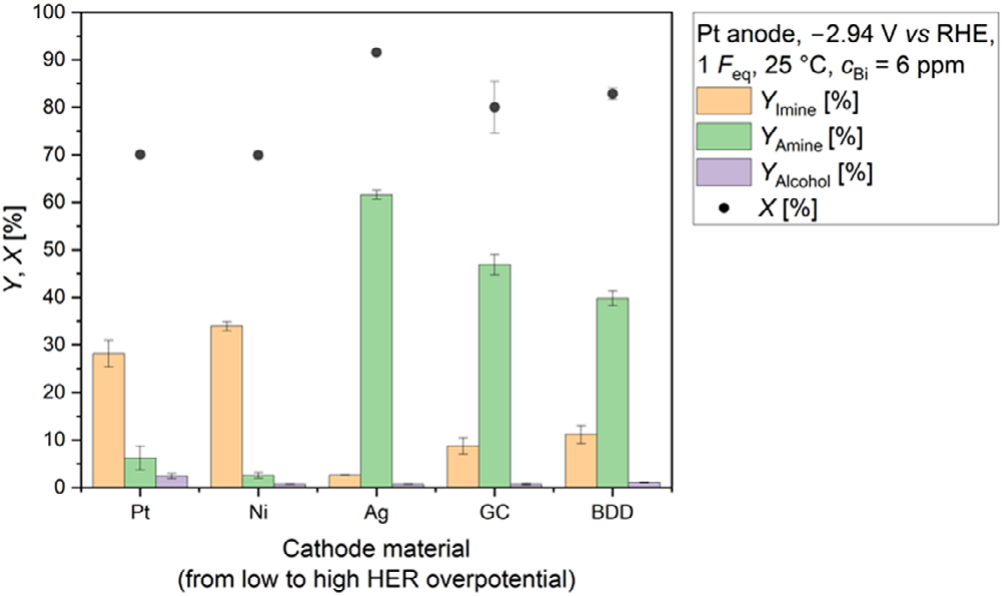
Effect of the cathode material in presence of 6 ppm Bi and at 25 °C on the electrochemical reductive amination of acetone with methylamine as nitrogen source. Yields of *N*‐methylpropan‐2‐imine (*Y*_Imine_), *N*‐methylpropan‐2‐amine (*Y*_Amine_), and isopropanol (*Y*_Alcohol_) are displayed. Furthermore, the conversion of acetone (*X*) is visualized. Carbon balance (CB): 65%–71%. Conditions: Ag||Pt; *E* = −2.94 V *vs* RHE; *F*_eq_ = 1; solvent: 0.5 M KH_2_PO_4_ (pH 8.3); substrates: acetone: 2.4 M, methylamine: 2.9 M; *T* = 25 °C; pH at 25 °C: 12.2; anolyte: 25% H_3_PO_4_; a N‐424 membrane; and 6 ppm Bi in the final substrate solution.

Nevertheless, a high amine yield in presence of trace amounts of Bi is not only defined by electrolysis temperature. Experiments have revealed that the choice of cathode material is also of great importance (Figure 6). At 25 °C and in presence of 6  ppm Bi, amine yields below 10% were achieved with good HER catalysts like Pt or Ni. In contrast, amine yields of 40% or more were obtained with cathode materials characterized by a high HER overpotential. This finding is in line with our previous study, in which we identified Pb as mediator in the electrochemical reductive amination [26]. However, at 25 °C and using Bi as mediator, the use of carbon based materials resulted in lower yields than applying Ag. One possibility is that the generation of bismuthane is more efficient on Ag than on glassy carbon (GC) or BDD, as the formation of volatile hydrides is expected to be the crucial step, but more research is required to support this statement and will be done in the future.

### 2.3 Outlook: Transfer of the Optimized Conditions to Different Substrate Combinations

**FIGURE 7:**
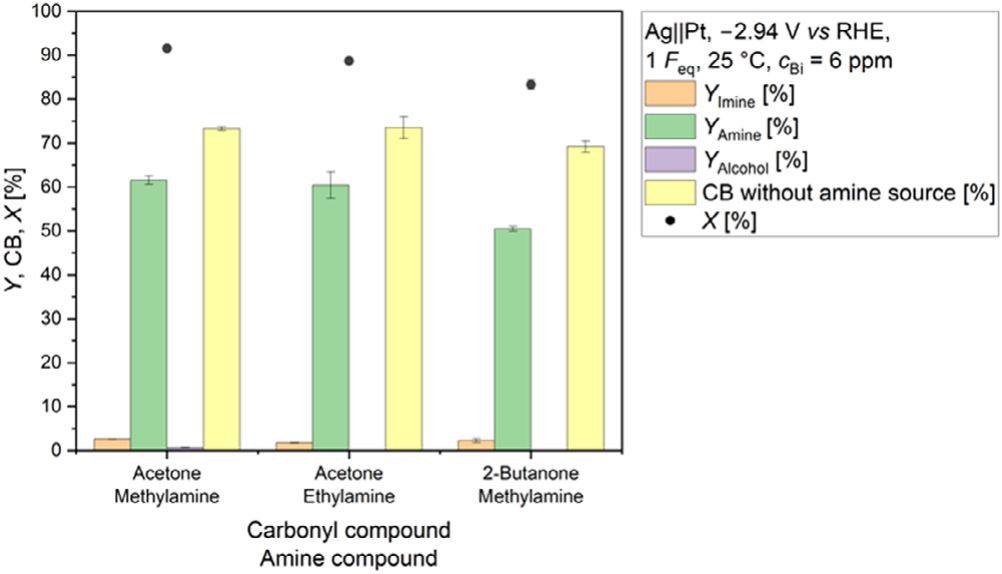
Electrochemical reductive amination of different substrate mixtures using Bi as mediator at 25 °C. Yields of the intermediates (*Y*_Imine_; *N*‐methylpropan‐2‐imine or *N*‐ethylpropan‐2‐imine or *N*‐methylbutan‐2‐imine), products (*Y*_Amine_; *N*‐methylpropan‐2‐amine or *N*‐ethylpropan‐2‐amine or *N*‐methylbutan‐2‐amine), and alcohols (*Y*_Alcohol_; isopropanol) are displayed. The side product 2‐butanol from 2‐butanone reduction was not observed. Furthermore, the conversion of carbonyl compound (*X*) and the carbon balance (CB) without taking the amine source into consideration are visualized. Conditions: Ag||Pt; *E* = −2.94 V *vs* RHE; *F*_eq_ = 1; solvent: 0.5 M KH_2_PO_4_ (pH 8.3); substrates: carbonyl: 2.4 M, amine: 2.9 M; *T* = 25 °C; pH at 25 °C: 12.2 (acetone + methylamine), 12.75 (acetone + ethylamine), 12.85 (2‐butanone + methylamine); anolyte: 25% H_3_PO_4_; a N‐424 membrane; and 6 ppm Bi in the final substrate solution.

Finally, we applied the optimized reaction conditions obtained based on the reference system with acetone and methylamine to the substrate combinations acetone and ethylamine (Scheme S1) as well as 2‐butanone and methylamine (Scheme S2). Thus, we demonstrate that the Bi‐mediated reductive amination is not limited to the combination of acetone and methylamine. In both cases, the respective amine was obtained in yields higher than 50% (Figure 7), illustrating the general feasibility and potential of Bi‐mediated electrochemical reductive amination. Currently, the system is limited to water‐soluble substrates, narrowing the range of testable substrates. Therefore, making water‐insoluble substrates accessible is the next big step and will be the topic of future studies.

## 3 Conclusion

In this study, the electrochemical reductive amination of acetone with methylamine as nitrogen source was investigated in the presence of various p‐block elements. It was found that not every element that can theoretically (electrochemically) form volatile hydrides can be successfully used as mediator for this type of reaction. Only Tl, Pb, and Bi, all elements of the sixth period, showed a reactivity. While Tl and Pb are highly toxic elements, Bi is considered to be non‐toxic [40, 41, 42, 43, 44]. Therefore, the electrochemical hydrogenation of *N*‐methylpropan‐2‐imine was optimized using Bi as additive by investigating the impact of additive concentration, reaction temperature, and cathode material on amine formation. In the presence of only 6 ppm Bi and using Ag as cathode material amine yields of more than 61% were obtained at room temperature. Finally, we applied the optimized conditions to substrate combinations of acetone and ethylamine as well as 2‐butanone and methylamine. The respective amines were obtained in yields higher than 50%. Overall, this study opens up novel possibilities for the non‐toxic and sustainable production of pharmaceuticals based on reductive amination.

## Conflicts of Interest

The authors declare no conflict of interest.

## Supporting information




**Supporting File 1**: The authors have cited additional references within the Supporting Information.

## Data Availability

The data that support the findings of this study are available via Zenodo: DOI: https://doi.org/10.5281/zenodo.17828688.
