# Supplementary material for: Traces of Elements in the Electrochemical Reductive Amination of Acetone: Uncovering Bi as Substitute for Pb
Source: Angew Chem Int Ed Engl. 2026 Feb 10;65(13):e21065. doi: 10.1002/anie.202521065 (PMC13007573; doi:10.1002/anie.202521065)
Supplement: Supplementary file 1 — Supporting File 1: The authors have cited additional references within the Supporting Information. [file ANIE-65-e21065-s001.docx]

Supplementary Information

Traces of Elements in the Electrochemical Reductive Amination of Acetone: Uncovering Bi as Substitute for Pb

Justus Kümper,^[a]^ Yani Guan,^[b]^ Simran Kumari,^[b]^ Sonja D. Mürtz,^[a]^ Philippe Sautet,*^[b,c]^ and Regina Palkovits*^[a,d,e]^

^a^ Chair of Heterogeneous Catalysis and Technical Chemistry
RWTH Aachen University
Worringerweg 2, 52074 Aachen (Germany).

^b^ Department of Chemical and Biomolecular Engineering
University of California, Los Angeles
5531-J Boelter Hall, Los Angeles, CA 90095 (USA).

^c^ Department of Chemistry and Biochemistry
University of California, Los Angeles
5531-J Boelter Hall, Los Angeles, CA 90095 (USA).

^d^ Institute for a Sustainable Hydrogen Economy (INW-2),

Forschungszentrum Jülich,

Marie-Curie-Str. 5, 52428 Jülich (Germany).

^e^ Max-Planck-Institute for Chemical Energy Conversion

Stiftstr. 34-36, 45470 Mülheim an der Ruhr (Germany).

* Author of correspondence: [palkovits@itmc.rwth-aachen.de](mailto:palkovits@itmc.rwth-aachen.de); [r.palkovits@fz-juelich.de](mailto:r.palkovits@fz-juelich.de); [sautet@ucla.edu](mailto:sautet@ucla.edu)

**Contents**

[Additional Results 3](#_Toc221274863)

[Experimental 8](#_Toc221274864)

[Nuclear magnetic resonance spectroscopy 9](#_Toc221274865)

[Analysis of the product solutions after electrolysis 9](#_Toc221274866)

[Investigation of imine formation as a function of temperature 10](#_Toc221274867)

[Assignment of the signals in the ^1^H-NMR spectrum 11](#_Toc221274868)

[Materials 13](#_Toc221274869)

[Calculation methods 14](#_Toc221274870)

[Reference 16](#_Toc221274871)

# Additional Results


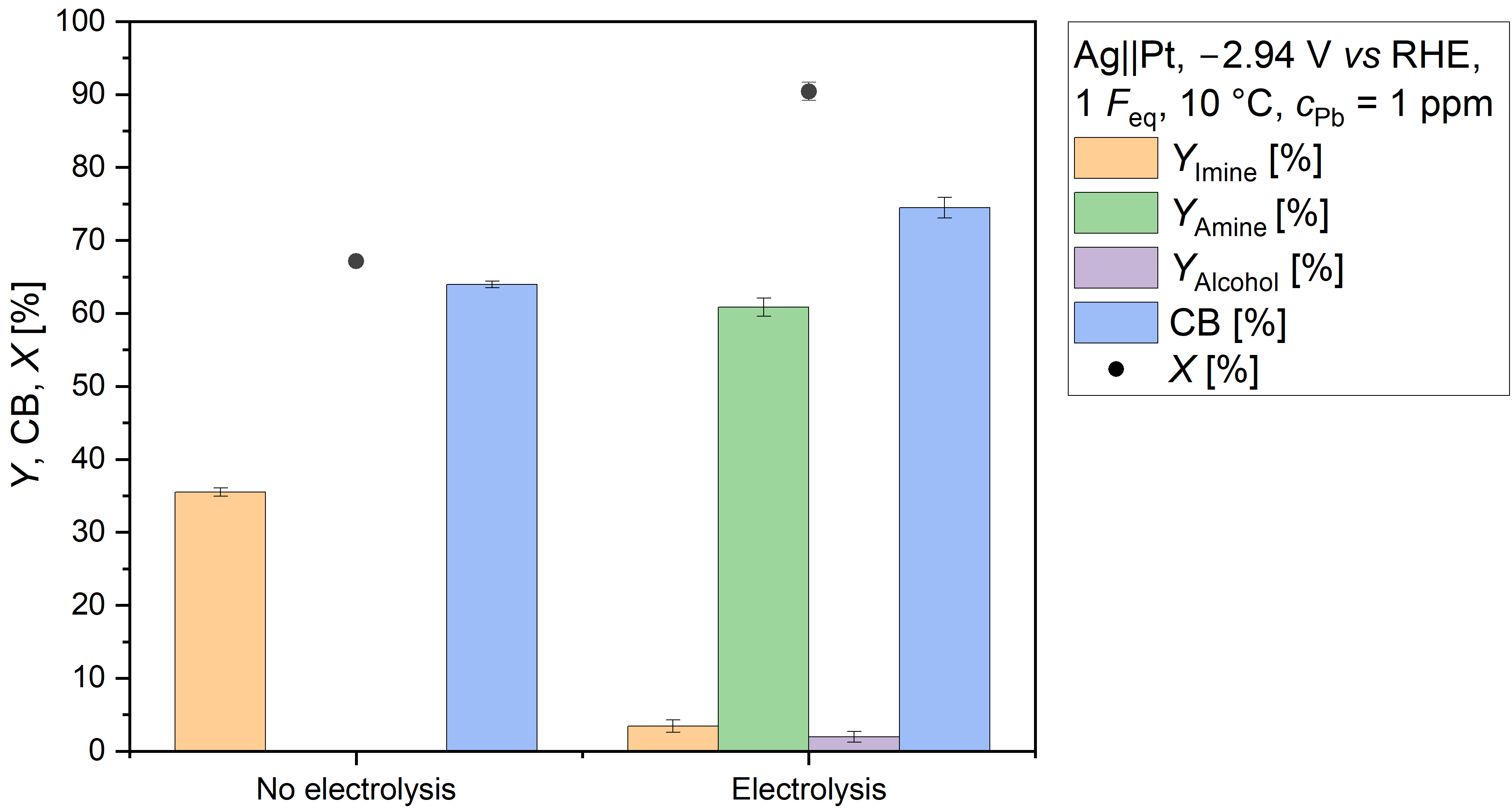


**Figure S1.** Effect of no electrolysis vs electrolysis on the yields of N‑methylpropan‑2‑imine (Y_Imine_), N‑methylpropan‑2‑amine (Y_Amine_), and isopropanol (Y_Alcohol_), the conversion (X) of acetone, and the carbon balance (CB). The electrolysis was completed after 2 hours, 54 minutes, and 25 seconds. The experiment in the absence of an electric current was terminated after the same amount of time as the experiment in presence of a current, and the reaction solution was analyzed as usual. The results verify that the electrolysis is not the reason for the strong decrease in CB, as in absence of a current a much lower CB was measured than in presence of a current. Conditions: Ag||Pt; E = −2.94 V vs RHE; F_eq_ = 1; solvent: 0.5 M KH_2_PO_4_ (pH 8.3); substrates: acetone: 2.4 M, methylamine: 2.9 M; T = 10 °C; pH at 10 °C: 12.9; anolyte: 25% H_3_PO_4_; and a N‑424 membrane. The electrolysis results based on 1 ppm Pb were already published in our previous work.^[1, 2]^


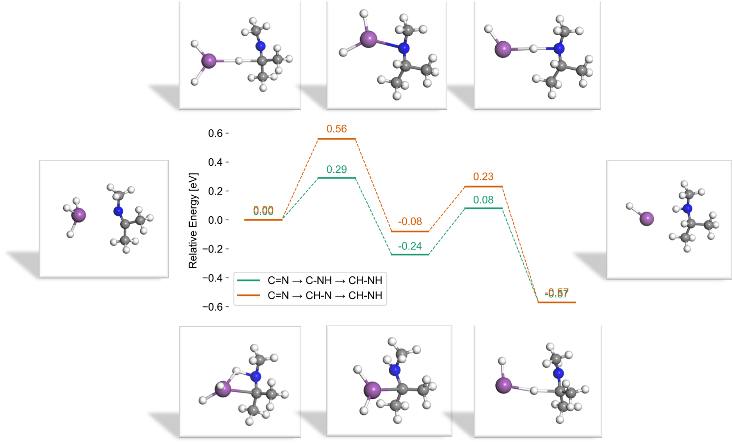


**Figure S2.** DFT-calculated energy profiles for two possible pathways of Bi-mediated imine hydrogenation: (i) C=N → C–NH → CH–NH (green) and (ii) C=N → CH–N → CH–NH (orange).


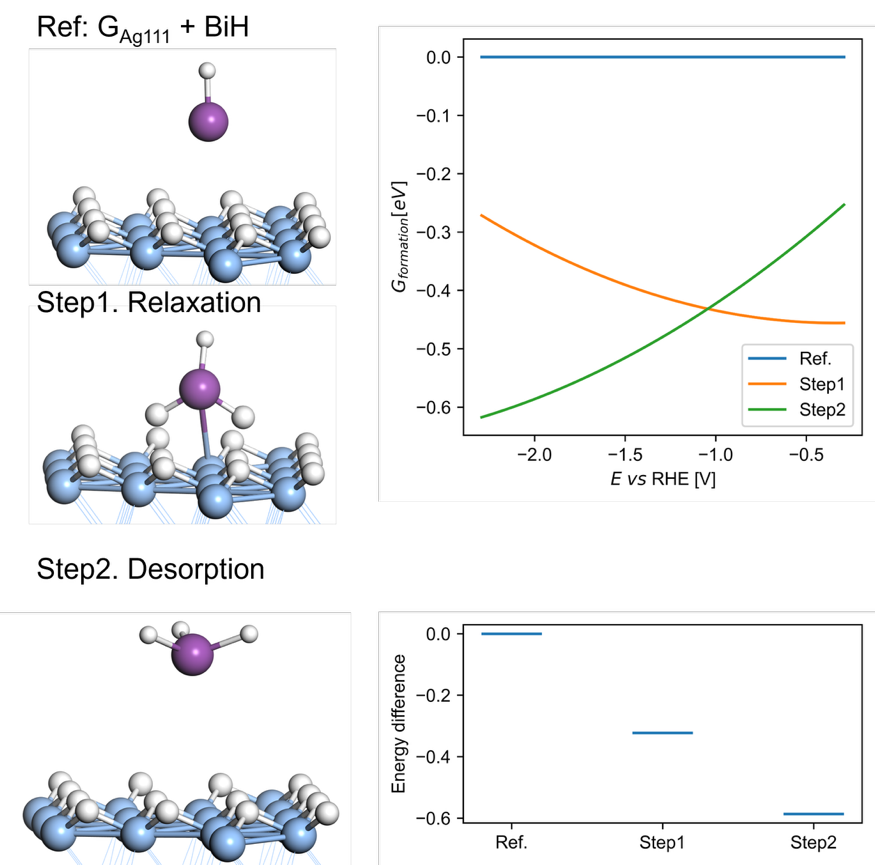


**Figure S3.** DFT analysis of Bi hydride species on Ag(111). Left: optimized structures of Bi adsorption and hydride intermediates. Right: (top) potential-dependent free energy diagram showing the relative stability of BiH_x_ intermediates compared to the reference state; (bottom) relative energy differences at −2 V *vs* RHE.


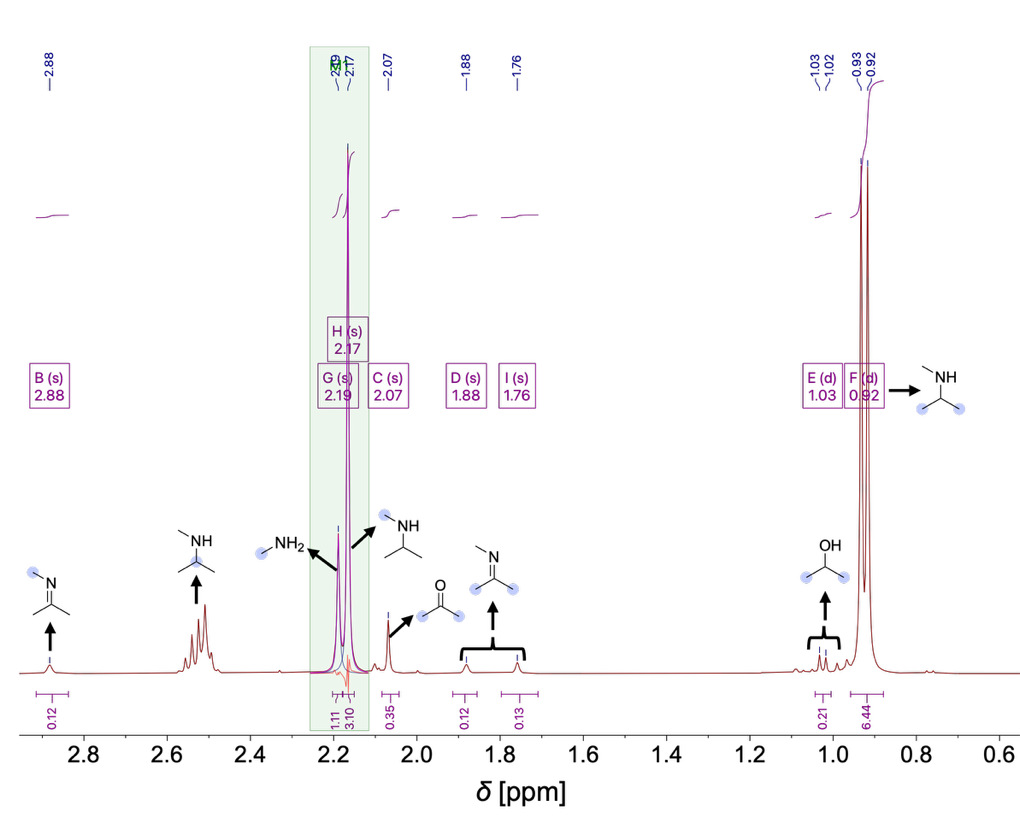


**Figure S4.** ^1^H‑NMR spectrum after potentiostatic electrolysis of an acetone‑methylamine mixture in presence of 1 ppm Pb. The signals are assigned to the substrates (acetone, methylamine), the intermediate N‑methylpropan‑2‑imine, and the products of the electrochemical hydrogenation (N‑methylpropan‑2‑amine, isopropanol). Conditions: Ag||Pt; E = −2.94 V vs RHE; F_eq_ = 1; solvent: 0.5 M KH_2_PO_4_ (pH 8.3); substrates: acetone: 2.4 M, methylamine: 2.9 M; T = 10 °C; pH at 10 °C: 12.9; anolyte: 25% H_3_PO_4_; a N‑424 membrane; and 1 ppm Pb in the final substrate solution. DMSO‑d_6_ was used as NMR solvent.


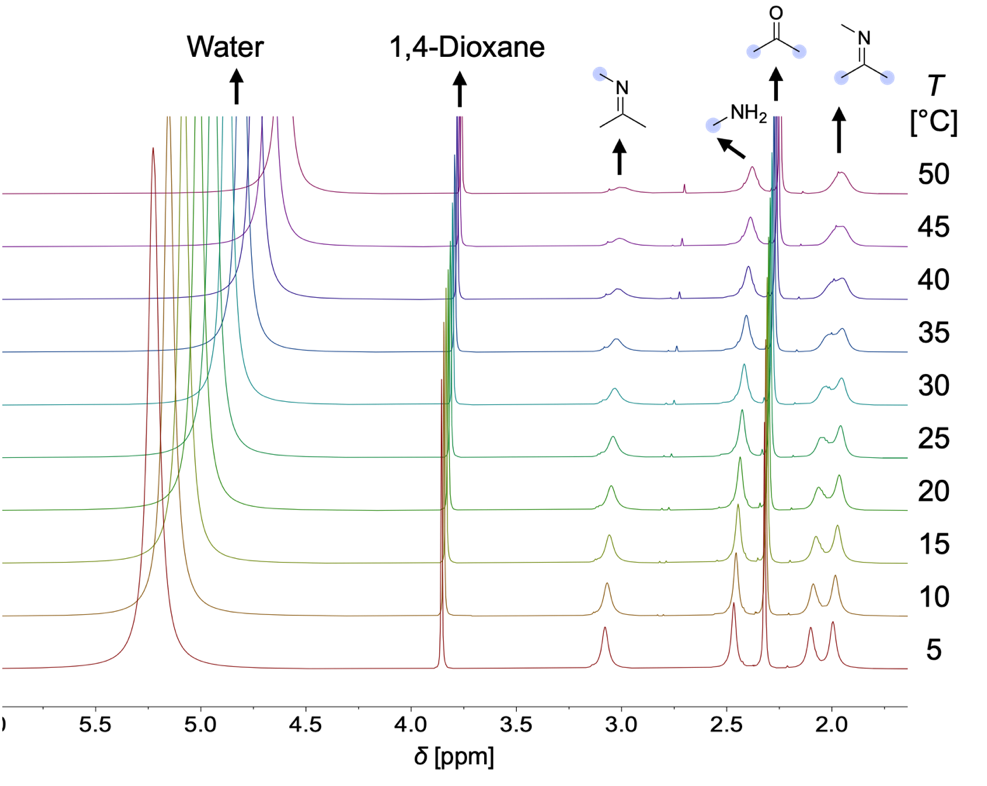


**Figure S5.** Stacked ^1^H-NMR spectra as a function of temperature to investigate the formation of N‑methylpropan‑2‑imine. Signals are assigned to the molecules and protons. Conditions: solvent: 0.5 M KH_2_PO_4_ (pH 8.3); substrates: acetone: 2.4 M, methylamine: 2.9 M. 1,4‑Dioxane was used as internal standard for quantification.

**Table S1.** Boiling points of acetone, methylamine (40% solution), N‑methylpropan‑2‑imine, and N‑methylpropan‑2‑amine.

| Compound | Boiling point [°C] |
| --- | --- |
| Acetone | 56^[3]^ |
| Methylamine (40% solution) | 48^[4]^ |
| *N*‑methylpropan‑2‑imine | 65^[5]^ |
| *N*‑methylpropan‑2‑amine | 49–51^[6]^ |


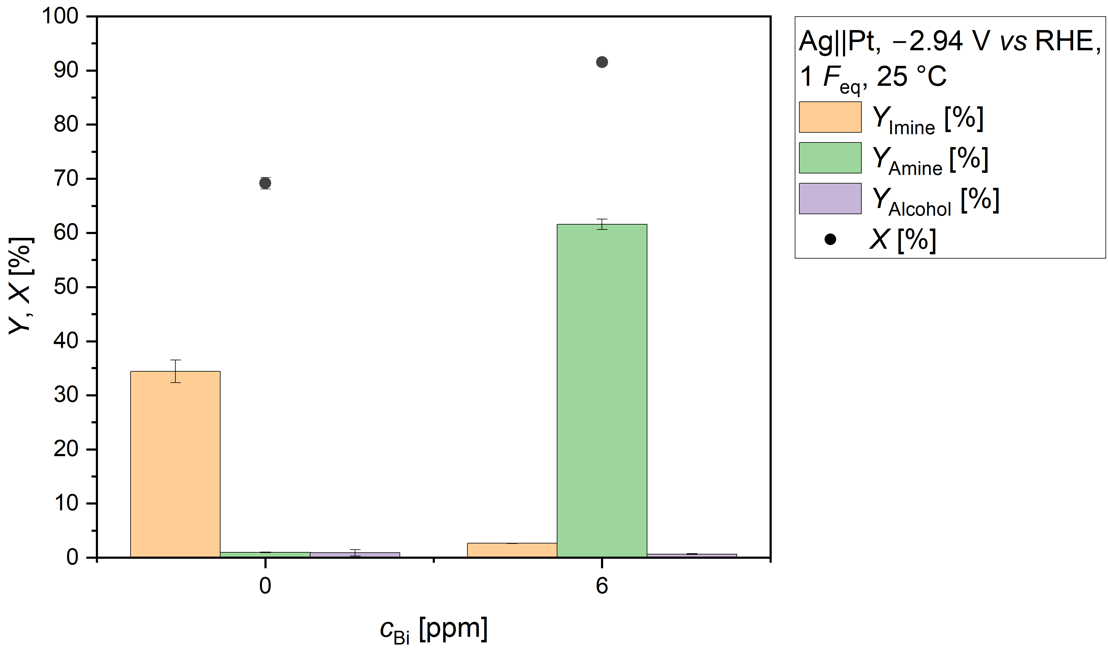
**Figure S6*.*** Effect of 6 ppm Bi on the electrochemical reductive amination of acetone at 25 °C. Yields of *N*‑methylpropan‑2‑imine (*Y*_Imine_), *N*‑methylpropan‑2‑amine (*Y*_Amine_), and isopropanol (*Y*_Alcohol_) are displayed. Furthermore, the conversion of acetone (*X*) and the carbon balance (CB) are visualized. Conditions: Ag||Pt; *E* = −2.94 V *vs* RHE; *F*_eq_ = 1; solvent: 0.5 M KH_2_PO_4_ (pH 8.3); substrates: acetone: 2.4 M, methylamine: 2.9 M; *T* = 25 °C; pH at 25 °C: 12.2; anolyte: 25% H_3_PO_4_; and a N‑424 membrane.


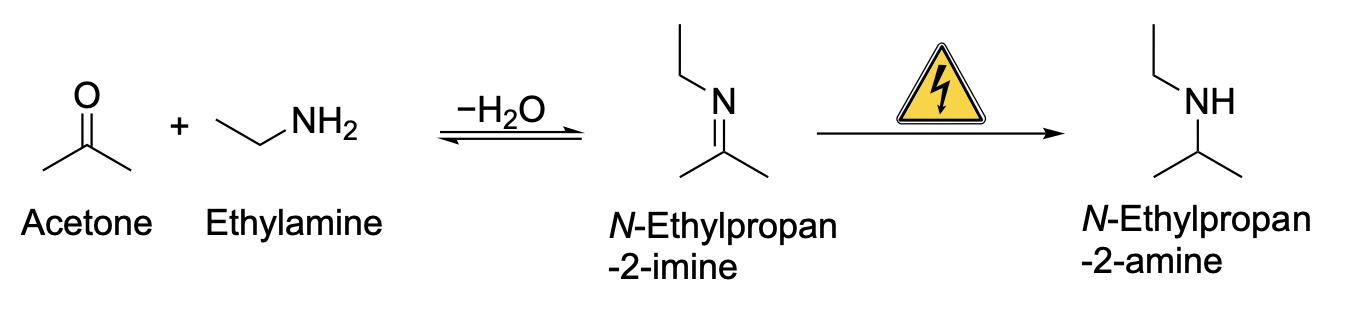


**Scheme S1.** Visualization of the imine intermediate and amine product, when acetone and ethylamine are used as substrates for the electrochemical reductive amination.


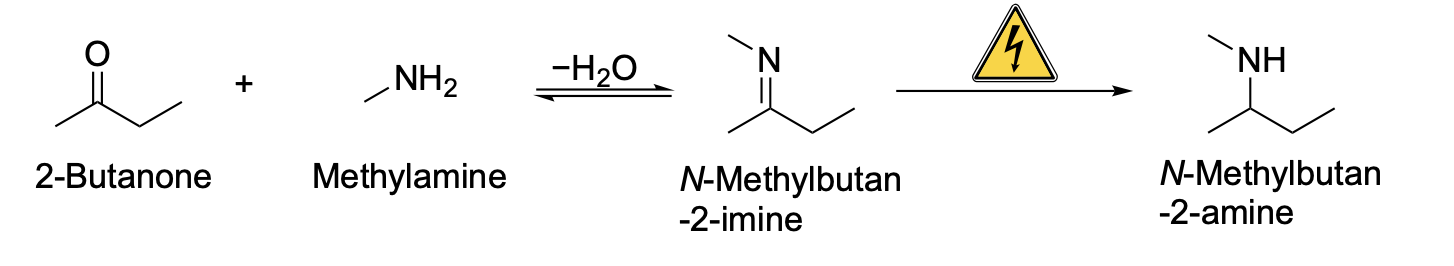


**Scheme S2.** Visualization of the imine intermediate and amine product, when 2-butanone and methylamine are used as substrates for the electrochemical reductive amination.


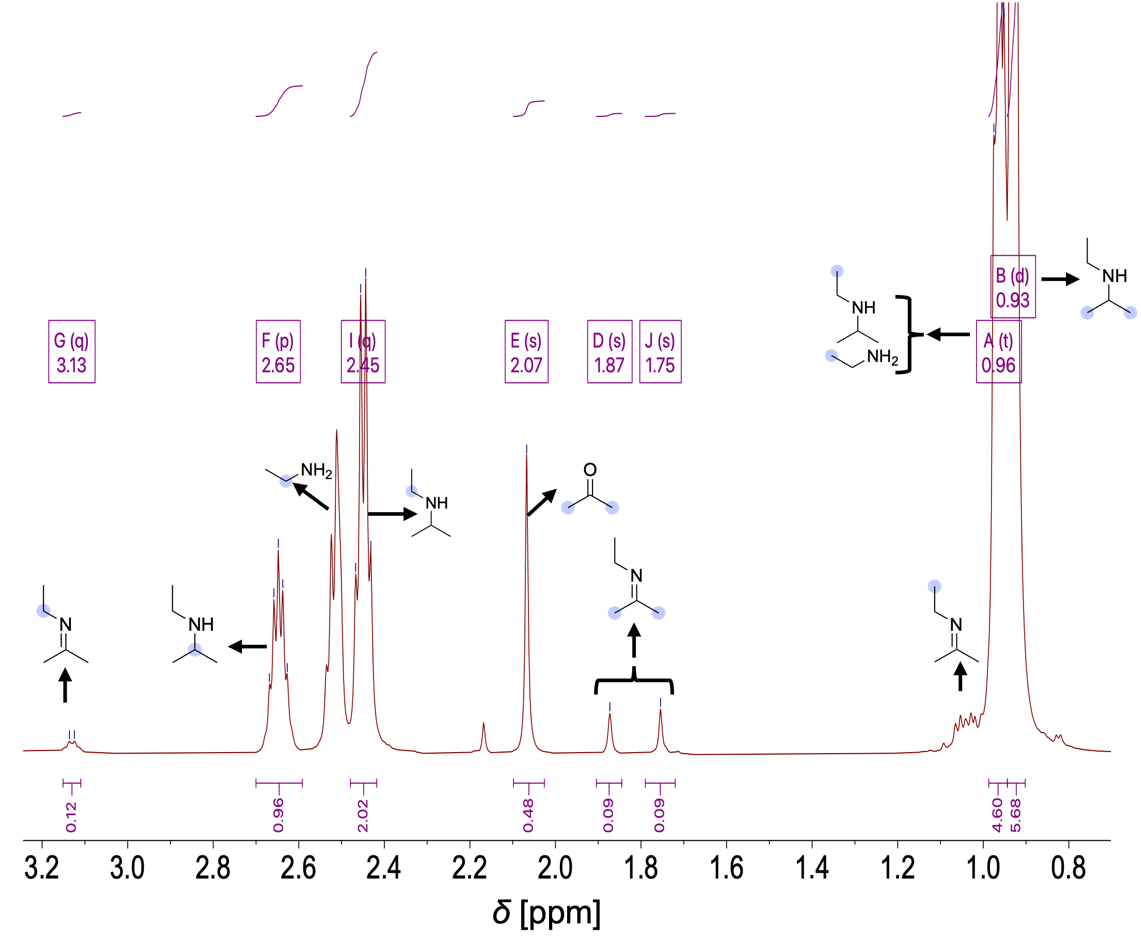


**Figure S7.** ^1^H‑NMR spectrum after potentiostatic electrolysis of an acetone‑ethylamine mixture in presence of 6 ppm Bi at 25 °C. The signals are assigned to the substrates (acetone, ethylamine), the intermediate N‑ethylpropan‑2‑imine, and the product of the electrochemical hydrogenation (N‑ethylpropan‑2‑amine). The side product isopropanol could not be identified. Conditions: Ag||Pt; E = −2.94 V vs RHE; F_eq_ = 1; solvent: 0.5 M KH_2_PO_4_ (pH 8.3); substrates: acetone: 2.4 M, ethylamine: 2.9 M; T = 25 °C; pH at 25 °C: 12.75; anolyte: 25% H_3_PO_4_; a N‑424 membrane; and 6 ppm Bi in the final substrate solution. DMSO‑d_6_ was used as NMR solvent.


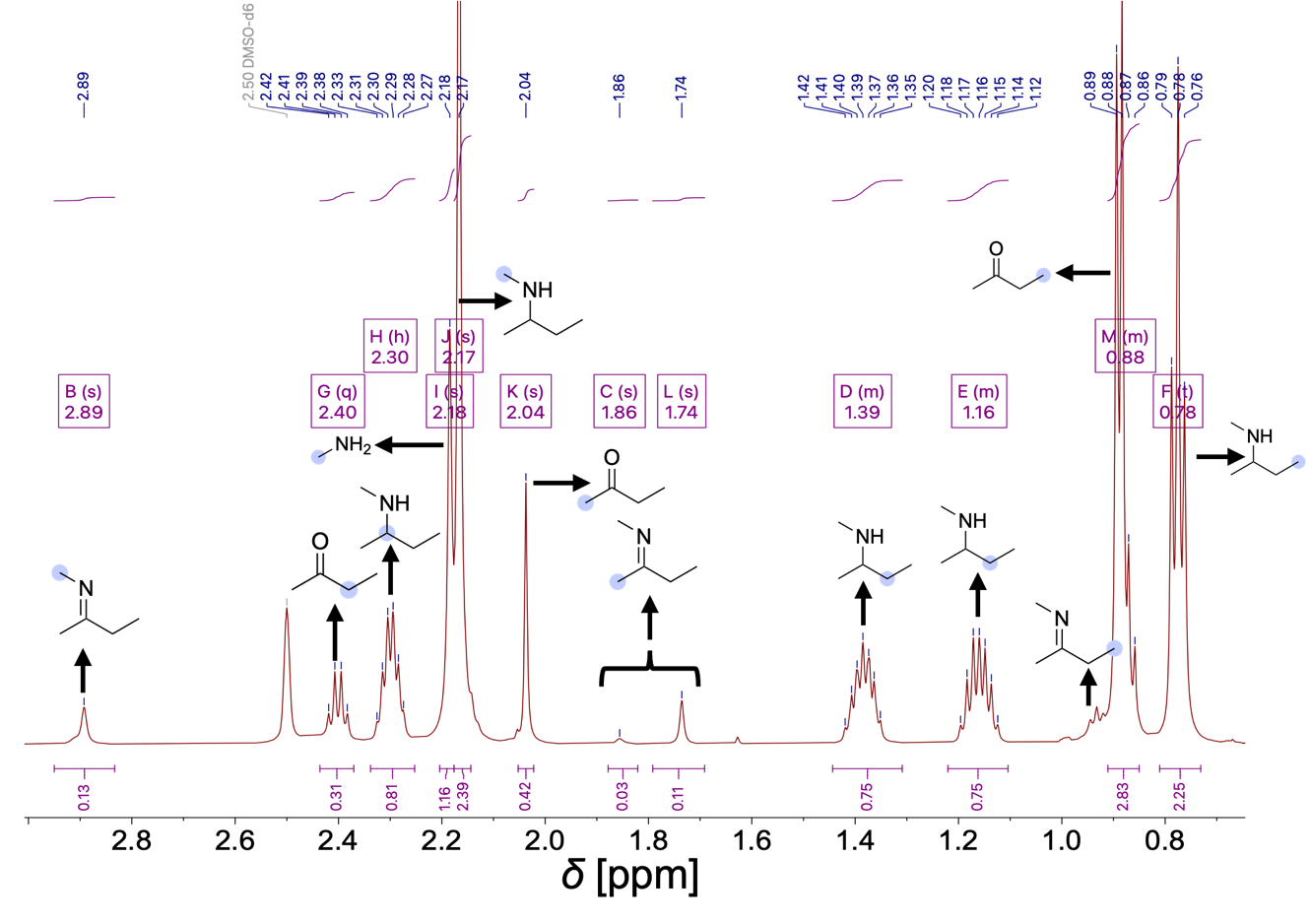


**Figure S8.** ^1^H‑NMR spectrum after potentiostatic electrolysis of a 2-butanone‑methylamine mixture in presence of 6 ppm Bi at 25 °C. The signals are assigned to the substrates (2-butanone, methylamine), the intermediate N‑methylbutan‑2‑imine, and the product of the electrochemical hydrogenation (N‑methylbutan‑2‑amine). The side product 2-butanol could not be identified. Conditions: Ag||Pt; E = −2.94 V vs RHE; F_eq_ = 1; solvent: 0.5 M KH_2_PO_4_ (pH 8.3); substrates: 2-butanone: 2.4 M, methylamine: 2.9 M; T = 25 °C; pH at 25 °C: 12.85; anolyte: 25% H_3_PO_4_; a N‑424 membrane; and 6 ppm Bi in the final substrate solution. DMSO‑d_6_ was used as NMR solvent.

# Experimental

The electrolysis experiments were performed in a custom‑built cell consisting of a stacked construction with cylindrical reaction chambers (r = 1 cm, h = 1 cm). The two reaction chambers (anode *vs* cathode compartment) were separated from each other by a *Nafion*-424 (N-424) membrane and closed from the other side with the respective electrode material. The temperature of the cathode compartment was set from the back side of the cathode using a cryostat (*Julabo* CORIO CD‑200F). A three‑electrode setup consisting of working (cathode), counter (anode), and reference electrode (Hg/HgO electrode (1 M KOH)) was used. Potentiostatic conditions were applied using a *Metrohm* Autolab PGSTAT 302N or PGSTAT204 potentiostat/galvanostat. Ag, Ni, Pt, glassy carbon (GC) or boron-doped diamond (BDD) were used as cathode materials. The Ag and Ni electrode were characterized by a mirror like surface, obtained by intensive polishing of the rolled metal material using a 1 µm alumina suspension (MicroPolish Alumina 1.0 µm from Buehler). Both electrodes were freshly polished before each electrolysis using the 1 µm alumina suspension. The cathode materials Pt, GC, or BDD were used as received. As anode, a Pt electrode was used.

The final reaction volume of 2 mL contained 2.4 M acetone (0.36 mL, 1 eq.) or 2-butanone (0.43 mL, 1 eq.), 2.9 M methylamine (1.2 eq., 40% methylamine solution: 0.50 mL) or ethylamine (1.2 eq., 65% ethylamine solution: 0.50 mL), the additive (As, Se, In, Sn, Sb, Te, Tl, Pb, or Bi), and the electrolyte that was used as solvent. The additive was added in dissolved form to the reaction solution. Each additive was tested separately, whereby its final concentration in the reaction solution was 1 ppm, unless otherwise stated. Normally, the additive was introduced into the reaction solution by using a 100 ppm standard solution of the respective element, prepared by dilution using deionized water. For As, Se, Sn, Sb, Te, and Bi, the respective 1000 ppm ICP standard solutions were used as the starting material. For In, Tl, and Pb, InCl_3_x4H_2_O, TlNO_3_, and Pb(NO_3_)_2_ were used as starting materials.

For final Bi concentrations between 1 ppm and 7 ppm, 100 ppm, 400 ppm, and 700 ppm standard solutions were used, which were prepared from a 1000 ppm Bi standard solution by dilution with deionized water. The 100 ppm Bi solution was used for the experiments with a final Bi concentration of 1 ppm and 2 ppm. The 400 ppm Bi solution was used for the experiments with 3 ppm, 4 ppm, 5 ppm, and 6 ppm Bi. And the 700 ppm Bi solution was used as the starting point for the experiments in which a final Bi concentration of 7 ppm was present.

An aqueous 0.5 M KH_2_PO_4_ solution (pH 8.3) was used as solvent for the reaction solutions. The volume of the solvent was adjusted to yield a final reaction volume of 2 mL. For the anode compartment, 2 mL 25% H_3_PO_4_ were used.

After transferring the solutions into the respective half‑cell compartment, the catholyte was adjusted to the desired reaction temperature within 18 min while stirring at 250 rpm. At 10 °C, the prepared reaction solution based on acetone and methylamine had a pH value of 12.9. In presence of 6 ppm Bi, the electrochemical reductive amination with these substrates was also performed at 5 °C, 15 °C, 20 °C, 25 °C, 35 °C, and 45 °C. The respective pH values are displayed in Table S2. When acetone and ethylamine or 2-butanone and methylamine were used as substrates, the electrolysis were performed at 25 °C. The pH of the substrate mixtures was 12.75 and 12.85 at 25 °C, respectively. The stirrer was turned off after 18 min of cooldown and cyclic voltammetry measurements were carried out with a scan rate of 50 mV s^−1^ between −1.12 V and 0.06 V *vs* RHE when acetone and methylamine were applied as substrates. The electrolysis experiments were performed at − 2.94 V *vs* RHE and stopped after 1 *F*_eq_ had passed (−926.3 C).

**Table S2.** pH values at the different temperatures for a reaction mixture based on acetone and methylamine containing 6 ppm Bi. Conditions: solvent: 0.5 M KH_2_PO_4_ (pH 8.3); substrates: acetone: 2.4 M, methylamine: 2.9 M.

| *T* [°C] | pH |
| --- | --- |
| 5 | 13.1 |
| 10 | 12.9 |
| 15 | 12.7 |
| 20 | 12.4 |
| 25 | 12.2 |
| 35 | 12.0 |
| 45 | 11.4 |

# Nuclear magnetic resonance spectroscopy

## Analysis of the product solutions after electrolysis

Yields, conversions, and carbon balances (CB) were determined by quantitative ^1^H‑NMR spectroscopy. As solvent, DMSO‑d_6_ (*δ*H: 2.50 ppm) was utilized. 1,3,5‑Trioxane (*δ*H: 5.07 ppm) and 1,4‑dioxane (*δ*H: 3.52 ppm) were used as internal standards for the cathode and anode solutions, respectively. The internal standard (~20 – 36 mg) was dissolved in DMSO‑d_6_ (0.8 mL) and frozen in the fridge in the meantime. After electrolysis, 0.2 mL of the product solution was added to the half‑thawed DMSO‑d_6_/internal standard mixture. The quantitative ^1^H‑NMR measurements were done with a *Bruker* Avance spectrometer (400 MHz) *Bruker* Avance III HD spectrometer (600 MHz) at room temperature with 16 scans in each case and setting the d1 time to 10 s. The NMR spectra were processed using apodization (0.3 Hz), zero filling (128 k (when 400 MHz device was used) or 256 k (when 600 MHz device was used)), phase correction (manually), and baseline correction (Bernstein polynomial fit (manually)). The quantification was done based on the absolute areas of the signals and using equation 1.

|  | $\text{n}_{\text{substance X}}\text{=}\text{n}_{\text{IS}}\text{∙}\frac{\text{H}_{\text{theo IS}}\text{∙}\text{A}_{\text{substance X}}}{\text{H}_{\text{substance X}}\text{∙}\text{A}_{\text{IS}}}\text{∙}\frac{\text{m}_{\text{product}}}{\text{m}_{\text{NMR}}}$ | 1 |
| --- | --- | --- |

*n*_substance X_: Amount of substance X after electrolysis [mol]

*n*_IS_: Amount of substance of the internal standard in the NMR sample solution [mol]

*H*_theo IS_: Number of protons the area of the used signal of the internal standard should theoretically represent [-]

*H*_substance X_: Number of protons the area of the used signal of substance X should theoretically represent [-]

*A*_substance X_: Measured area of the used signal of substance X [-]

*A*_IS_: Measured area of the used signal of the internal standard [-]

*m*_product_: Mass of the product solution after electrolysis [g]

*m*_NMR_: Mass of the product solution used for the NMR analysis [g]

## Investigation of imine formation as a function of temperature

The investigation of imine formation as a function of temperature was carried out using quantitative ^1^H-NMR spectroscopy (Figure S5). The reaction solution based on 2.4 M acetone (0.71 mL, 1 eq.), 2.9 M methylamine (1.2 eq., 40% methylamine solution: 1.00 mL), and the electrolyte, which acted as the solvent. An aqueous 0.5 M KH_2_PO_4_ solution (2.29 mL) (pH 8.3) was used as electrolyte. In addition, 1,4*‑*dioxane (0.18 mL) was added to the reaction mixture as an internal standard to ultimately quantify the yields (*Y*), conversions (*X*), and carbon balances (CB). It was assumed that the 1,4*‑*dioxane had no effect on the reaction between acetone and methylamine. This reaction mixture was then transferred into an NMR tube with a screw cap (*Wilmad*, 300 MHz, diameter 5 mm). Benzene-d_6_ was transferred into a *Wilmad* coaxial insert. This coaxial insert was then inserted into the NMR tube with screw cap, which contained the reaction solution with internal standard (Figure S9). After reaching the target temperature (5 °C, 10 °C, 15 °C, 20 °C, 25 °C, 30 °C, 35 °C, 40 °C, 45 °C, and 50 °C), the measurement was started after a waiting period of 10 minutes for equilibration. Preliminary tests showed that equilibrium was reached after 10 min. Quantitative ^1^H*‑*NMR measurements were performed with a *Bruker* Avance III HD spectrometer (600 MHz) with 16 scans each and a d1 time of 10 s. The resulting NMR spectra were processed as follows using MNova software (*Mestrelab Research*): apodization (0.3 Hz), zero*‑*point filling (128 k), phase correction (manual), and baseline correction (Bernstein polynomial fit (manual)). Quantification was based on the area of the signals and equation 1. ^1^H*‑*NMR measurements were performed by Ines Bachmann-Remy (ITMC, RWTH Aachen University).


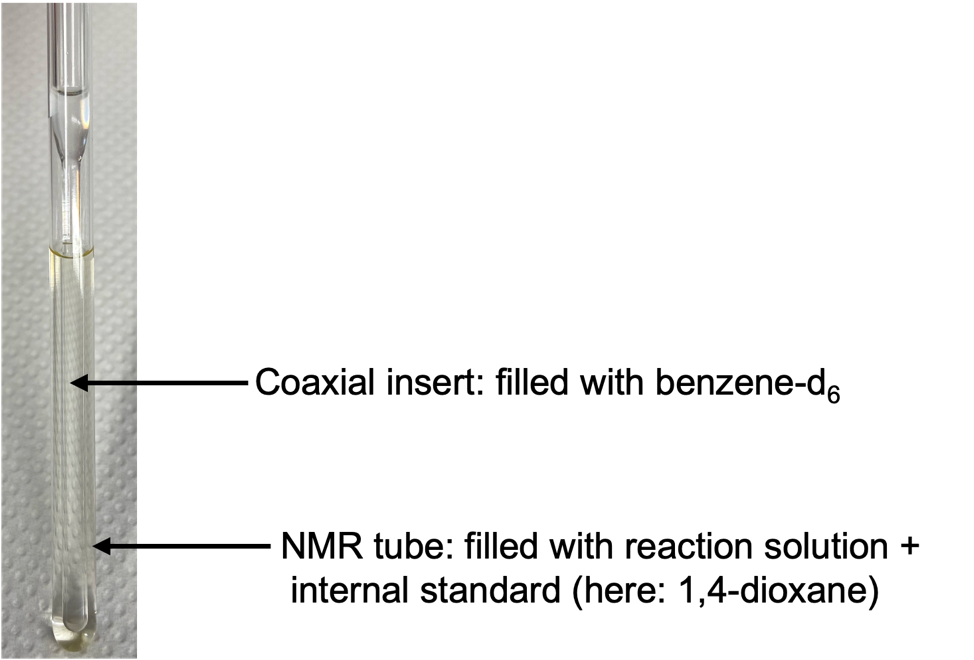


**Figure S9.** NMR tube with coaxial insert: set-up used to investigate imine formation as a function of temperature.

## Assignment of the signals in the ^1^H-NMR spectrum

Abbreviations: s = singlet, d = doublet, t = triplet, q = quartet, p = pentet, h = hextet, hept = heptet, m = multiplet.

***Reductive amination of acetone with methylamine*:**

Acetone: ^1^H‑NMR (400 MHz, DMSO‑d_6_) *δ* [ppm] = **2.06** (s, 6H).

Methylamine: ^1^H‑NMR (400 MHz, DMSO‑d_6_) *δ* [ppm] = **2.17** (s, 3H).

*N*‑Methylpropan‑2‑imine: ^1^H‑NMR (400 MHz, DMSO‑d_6_) *δ* [ppm] = **2.87** (s, 3H), **1.88** (s, 3H), **1.76** (s, 3H).

*N*‑Methylpropan‑2‑amine: ^1^H‑NMR (400 MHz, DMSO‑d_6_) *δ* [ppm] = 2.58–2.45 (m, 1H), **2.16** (s, 3H), **0.92** (d, *J* = 6.3 Hz, 6H).

Isopropanol: ^1^H‑NMR (400 MHz, DMSO‑d_6_) *δ* [ppm] = 3.76 (hept, *J* = 6.1 Hz, 1H), **1.02** (d, *J* = 6.1 Hz, 6H).

Signals used for the calculation of yield, conversion, and CB are printed in **bold**. Slight changes in the chemical shifts can be due to the use of different solutions (before *vs* after electrolysis).

***Reductive amination of acetone with ethylamine*:**

Acetone: ^1^H‑NMR (600 MHz, DMSO‑d_6_) *δ* [ppm] = **2.07** (s, 6H).

Ethylamine: ^1^H‑NMR (600 MHz, DMSO‑d_6_) *δ* [ppm] = 2.51 (q, *J* = 7.1 Hz, 2H), 0.96 (t, *J* = 7.1 Hz, 3H).

*N*‑Ethylpropan‑2‑imine: ^1^H‑NMR (600 MHz, DMSO‑d_6_) *δ* [ppm] = 3.13 (q, *J* = 7.4 Hz, 2H), **1.87** (s, 3H), **1.75** (s, 3H), 1.05 (t, *J* = 7.3 Hz, 3H).

*N*‑Ethylpropan‑2‑amine: ^1^H‑NMR (600 MHz, DMSO‑d_6_) *δ* [ppm] = **2.65** (p, *J* = 6.3 Hz, 1H), **2.45** (q, *J* = 7.1 Hz, 2H), 0.96 (t, *J* = 7.3 Hz, 3H), **0.93** (d, *J* = 6.8 Hz, 6H).

Signals used for the calculation of yield, conversion, and CB are printed in **bold**. The CB was calculated without taking ethylamine and isopropanol into consideration, as their signals overlapped with other signals. Slight changes in the chemical shifts can be due to the use of different solutions (before *vs* after electrolysis).

***Reductive amination of 2-butanone with methylamine*:**

2-Butanone: ^1^H‑NMR (600 MHz, DMSO‑d_6_) *δ* [ppm] = **2.40** (q, *J* = 7.3 Hz, 2H), **2.04** (s, 3H), 0.87 (t, *J* = 7.3 Hz, 3H).

Methylamine: ^1^H‑NMR (600 MHz, DMSO‑d_6_) *δ* [ppm] = 2.19 (s, 3H).

*N*‑Methylbutan‑2‑imine: ^1^H‑NMR (600 MHz, DMSO‑d_6_) *δ* [ppm] = **2.90** (s, 3H), 2.15 (q, *J* = 7.6 Hz, 2H), **1.85** (s, 3H), **1.73** (s, 3H), **0.93** (t, *J* = 7.5 Hz, 3H).

*N*‑Methylbutan‑2‑amine: ^1^H‑NMR (600 MHz, DMSO‑d_6_) *δ* [ppm] = **2.30** (h, *J* = 6.0 Hz, 1H), 2.17 (s, 3H), **1.44–1.32** (m, 1H), **1.22–1.09** (m, 1H), **0.77** (t, *J* = 7.5 Hz, 3H).

Signals used for the calculation of yield, conversion, and CB are printed in **bold**. The CB was calculated without taking methylamine into consideration, as their signals overlapped with other signals. 2-Butanol was not observed in the spectrum of the product solution. Slight changes in the chemical shifts can be due to the use of different solutions (before *vs* after electrolysis).

# Materials

The chemicals used in this work are listed in Table S3 below. They were used without any purification.

**Table S3.** Manufacture and purity of the chemical/material used in the experiments.

| Substance/Material | Manufacture | Purity |
| --- | --- | --- |
| 1,3,5-Trioxane | Aldrich | 99% |
| 1,4-Dioxane | Emsure | 99.50% |
| 2-Butanone | Sigma-Aldrich | ≥ 99,5 % |
| Acetone | Chemsolute | 99.50% |
| Antimony standard solution for ICP (1000 ppm Sb in 20% HCl) | Alfa | - |
| Arsenic standard solution for ICP (1000 ppm As in HNO_3_) | Fluka Chemie AG | 1000 ppm ±0.3% |
| Benzene-d_6_ | Deutero | 99.00% |
| Bismuth standard solution for ICP (1000 ppm Bi in HNO_3_ (3%)) | Roth | Starting material 99.991 % |
| Dimethylsulfoxide-d_6_ | Deutero | 99.80% |
| Ethylamine | Sigma-Aldrich | 65 % |
| Indium(III) chloride tetrahydrate | Aldrich / Merck KGaA | 99.999 % |
| Lead(II) nitrate | Roth | ≥99% |
| Methylamine | Merck | 40% |
| MicroPolish Alumina 1.0 µm | Buehler |  |
| Monopotassium phosphate | Fluka | 99% |
| *Nafion* N-424 | Ion Power |  |
| Phosphoric acid | Merck | 85% |
| Platinum | Evochem | 99.95% |
| Potassium hydroxide | Chemsolute | 85% |
| Selenium standard solution for ICP (1000 ppm Se in HNO_3_ (~0.5 M)) | Fluka Chemie AG | 1000 ppm ±0.3% |
| Silver | Chempure | 99.90% |
| Tellurium standard solution for ICP (1000 ppm Te in HNO_3_ (2%)) | Roth | Starting material 99.96% |
| Thallium(I) nitrate | Sigma-Aldrich/Merck | 99.999% |
| Tin standard solution for ICP (1000 ppm Sn in HCl (7%)) | Merck KGaA | - |

# Calculation methods

The Vienna Ab initio Simulation Package (VASP) was employed for all periodic DFT calculations^[7]^. The Perdew-Burke-Ernzerhof (PBE) functional within the generalized gradient approximation (GGA) was used for the exchange-correlation, along with the dDsC dispersion correction to account for van der Waals interactions^[8, 9]^. The energy cutoff was set to 400 eV. Interactions between atomic cores and electrons were modeled using the projector augmented wave (PAW) method^[10]^. Initial geometry optimizations were performed in vacuum until the forces on each atom were below 0.01 eV/Å and the total energy converged to less than 10⁻^6^ eV. Subsequently, geometry optimizations using implicit solvation via VASPsol^[11, 12]^ were conducted, with convergence criteria of 0.02 eV/Å for forces and 10⁻^6^ eV for energy.

A 3-layer (4 × 4) supercell of Ag(111) termination was used, where the bottom one layer was fixed. A vacuum slab of 15 Å thickness was added in the *Z* direction. When it comes to solvation optimization and Surface Charging, all structures are symmetrized and the box thickness in the *Z* direction is 60 Å for the implicit solvation region. To be specific, five layers were used with water layers and/or adsorbates on both sides. The Brillouin zone was sampled using 5 × 5 × 1 Gamma-centered k-point grids for structure optimization. The model with three water layers is constructed layer by layer to achieve fully equilibrium within and between layers.

4.1.1 GCDFT simulations of solvated models on Ag(111)

The grand canonical free energy was evaluated by grand canonical density functional theory (GCDFT) calculations, which is a surface charging technique. Details can be found in our previous work, and here we summarize the key points^[13-16]^. The net charge of the surface 𝑛_surface_ is obtained as:

$$n_{surface}=N_{surface}-N_{surface,neutral}$$

Where $N_{surface}$ and $N_{surface,neutral}$ is the number of electrons on the surface and the number of electrons in the neutral state. DFT energy for the charged surface is obtained as:

$$E_{surface}=E_{surface,raw}+\epsilon_{fermishift}n_{surface}$$

Where $E_{surface,raw}$ is the raw electronic energy of the surface and $\epsilon_{fermishift}n_{surface}$is the correction term accounting for the difference ($\epsilon_{fermishift}$) in the reference energy of the electron between the “internal” reference level and vacuum. Then, the grand canonical electronic energy of a surface model, $\Omega\left( E \right)$ is obtained as:

$$\Omega\left( E \right)=E_{surface}-n_{surface}\mu_{electron}$$

Where $\mu_{electron}$is the chemical potential of an electron, which is defined as:

$$\mu_{electron}={qE}_{vac}=-eE_{vac}$$

Where $E_{vac}$ is the potential of the system with reference to the vacuum level and *q* is the charge of an electron. The potential of the system with reference to the vacuum can be determined by two components, the Fermi level ($\epsilon_{F}$) with reference to the “internal” zero energy reference and the Fermi shift which is the difference between the “internal” energy reference and the vacuum level:

$$-eE_{vac}=\epsilon_{F}+\epsilon_{fermishift}$$

For the metallic systems, the potential-dependent grand canonical energy, $\Omega\left( E \right)$, exhibits a quadratic behaviour around the potential of zero charge (*E*_0_) in the vacuum scale:

$$\Omega\left( E \right)=\Omega\left( E_{0} \right)-\frac{1}{2}C(E-E_{0})^{2}$$

Where *C* is the capacitance of the surface. The potential of the system with respect to the standard hydrogen electrode (SHE) ($E_{SHE}$) can be converted from $E_{vac}$as:

$$E_{SHE}=E_{vac}-4.44$$

The linearized Poisson Boltzmann implicit solvation model implemented in VASPsol^[11]^ is used to represent the polarizable electrolyte region. The dielectric constant of water, 78.4, and the Debye screening length corresponding to 0.1 M concentration of electrolytes, 9.6 Å, were used. The surface slab is symmetrized along the z-axis to avoid asymmetric potential in the implicit solvation region. Here the implicit solvent thickness is set to 60 Å for the symmetrized slab.

4.1.2 Homogeneous hydrogenation reaction in solution calculations

To model the solution-phase reaction environment, we constructed a cubic simulation box with dimensions of 25 Å × 25 Å × 25 Å. Implicit solvation was incorporated through the VASPsol framework to approximate electrolyte conditions, thereby providing a more realistic representation of the reaction medium. This setup enables the simulation of homogeneous reaction dynamics between BiH₃ and imine. In this process, BiH_3_ serves as a hydrogen donor, driving the hydrogenation of imine to form the corresponding amine, while undergoing partial dehydrogenation to yield BiH_1_ as a byproduct. This computational framework offers mechanistic insight into the pathway and energetics of imine hydrogenation under electrolyte-like conditions. To determine the associated reaction energy barriers for homogeneous hydrogenation in solution, the CI-NEB method was employed.

# Reference

[1] J. Kümper, S. D. Mürtz, Y. Guan, S. Kumari, P. J. C. Hausoul, N. Kurig, P. Sautet, R. Palkovits, Metallic Impurities in Electrolysis: Catalytic Effect of Pb Traces in Reductive Amination and Acetone Reduction, *Angew. Chem. Int. Ed. Engl.* **2024**, *63*, e202411532.

[2] J. Kümper, S. D. Mürtz, Y. Guan, S. Kumari, P. J. C. Hausoul, N. Kurig, P. Sautet, R. Palkovits, **2024**, *Dataset for the publication: Metallic Impurities in Electrolysis: Catalytic Effect of Pb Traces in Reductive Amination and Acetone Reduction*, Zenodo, Version v1, 10.5281/zenodo.13375716.

[3] Sigma-Aldrich Chemie GmbH, *Safety Data Sheet of Acetone*, last access: 12/01/25, <https://www.sigmaaldrich.com/DE/en/sds/sigald/179124?userType=undefined>.

[4] Carl Roth GmbH + Co. KG, *Methylamine 40 % in water, for synthesis*, 09/21/24, last access: 12/01/25, <https://www.carlroth.com/medias/SDB-1E51-MT-EN.pdf?context=bWFzdGVyfHNlY3VyaXR5RGF0YXNoZWV0c3wzNTAxMTR8YXBwbGljYXRpb24vcGRmfGFHRXdMMmhrTmk4NU1UWTVPVFkwTmpNd01EUTJMMU5FUWw4eFJUVXhYMDFVWDBWT0xuQmtaZ3xkZTZkMzYyM2I5MWIyZjU1Yzk0MGQxYjRlYTBjYjFkM2NjOGU5ZjY3Y2M0ZGNkMTYwMWIzNGE4ZjA3MDM1OWY5>.

[5] G. A. Olah, P. Kreienbuehl, Stable carbonium ions. L. Protonated imines, *J. Am. Chem. Soc.* **1967**, *89*, 4756-4759.

[6] M. A. Shaik, H. Oelschläger, D. Rothley, Notiz zur Darstellung des N-Methylisopropylamins, *Arch. Pharm.* **1981**, *314*, 644-646.

[7] G. Kresse, J. Furthmüller, Efficiency of ab-initio total energy calculations for metals and semiconductors using a plane-wave basis set, *Comput. Mater. Sci.* **1996**, *6*, 15-50.

[8] J. P. Perdew, K. Burke, M. Ernzerhof, Generalized Gradient Approximation Made Simple, *Phys. Rev. Lett.* **1996**, *77*, 3865-3868.

[9] S. Gautier, S. N. Steinmann, C. Michel, P. Fleurat-Lessard, P. Sautet, Molecular adsorption at Pt(111). How accurate are DFT functionals?, *Phys. Chem. Chem. Phys.* **2015**, *17*, 28921-28930.

[10] G. Kresse, D. Joubert, From ultrasoft pseudopotentials to the projector augmented-wave method, *Phys. Rev. B* **1999**, *59*, 1758-1775.

[11] K. Mathew, V. S. C. Kolluru, S. Mula, S. N. Steinmann, R. G. Hennig, Implicit self-consistent electrolyte model in plane-wave density-functional theory, *J. Chem. Phys.* **2019**, *151*, 234101.

[12] K. Mathew, R. Sundararaman, K. Letchworth-Weaver, T. A. Arias, R. G. Hennig, Implicit solvation model for density-functional study of nanocrystal surfaces and reaction pathways, *J. Chem. Phys.* **2014**, *140*, 084106.

[13] X. Fu, D. Cheng, C. Wan, S. Kumari, H. Zhang, A. Zhang, H. Huyan, J. Zhou, H. Ren, S. Wang, Z. Zhao, X. Zhao, J. Chen, X. Pan, P. Sautet, Y. Huang, X. Duan, Bifunctional Ultrathin RhRu(0.5) -Alloy Nanowire Electrocatalysts for Hydrazine-Assisted Water Splitting, *Adv. Mater.* **2023**, *35*, e2301533.

[14] S. Kumari, T. Masubuchi, H. S. White, A. Alexandrova, S. L. Anderson, P. Sautet, Electrocatalytic Hydrogen Evolution at Full Atomic Utilization over ITO-Supported Sub-nano-Pt(n) Clusters: High, Size-Dependent Activity Controlled by Fluxional Pt Hydride Species, *J. Am. Chem. Soc.* **2023**, *145*, 5834-5845.

[15] S. Kumari, P. Sautet, Elucidation of the Active Site for the Oxygen Evolution Reaction on a Single Pt Atom Supported on Indium Tin Oxide, *J. Phys. Chem. Lett.* **2023**, *14*, 2635-2643.

[16] D. Cheng, Z. Wei, Z. Zhang, P. Broekmann, A. N. Alexandrova, P. Sautet, Restructuring and Activation of Cu(111) under Electrocatalytic Reduction Conditions, *Angew. Chem. Int. Ed. Engl.* **2023**, *62*, e202218575.
